# A critical narrative analysis of psychiatrists’ engagement with psychosis as a contentious area

**DOI:** 10.1177/0020764020934516

**Published:** 2020-06-26

**Authors:** Therese O’Donoghue, Jon Crossley

**Affiliations:** Department of Neuroscience, Psychology and Behaviour, George Davies Centre, University of Leicester, Leicester, UK

**Keywords:** Psychosis, critical psychiatry, critical narrative analysis

## Abstract

**Background:**

Psychosis, characterised by altered perceptions or interpretations of reality, remains a contested area. Lately, perspectives and conceptualisations of psychosis that have traditionally been more peripheral have gained greater recognition. Both the British Psychological Society and Critical Psychiatry Network have highlighted some contentious areas in recent publications.

**Aims:**

The aim was to use critical narrative analysis to consider what facilitates and inhibits medical professionals with clinical experience of psychosis to engage with the topic of psychosis as a contentious area.

**Method:**

Semi-structured interviews were conducted with 12 medical professionals, who were at trainee or qualified level with a minimum of 6 months’ clinical experience within psychiatry, across three Trusts within the United Kingdom. This purposive sample had a diverse range of perspectives regarding psychosis. Critical narrative analysis comprising six distinct stages, informed the analysis.

**Results:**

Participants positioned themselves broadly within one of three groups: biological psychiatrists, critical psychiatrists and those more conflicted. Narrative analysis was undertaken for each participant before being integrated for this article. The research highlighted several factors which either limit or facilitate opportunities within the psychiatric profession to engage with a plurality of views regarding psychosis. These included the significance of power and hierarchy within the profession, the role of dialogue and the prevalence of reflexivity within the profession.

**Conclusion:**

A pattern was identified of psychiatrists generally associating with like-minded others and not engaging with wider evidence regarding psychosis, partly as a result of the inherent threats to the power and hierarchy of the profession. This led to new ideas being widely unknown or undervalued, potentially to the disservice of clinical practice.

## Introduction

The conceptualisation of psychosis is inevitably influenced by theories of etiology. Within psychiatry, although there is a diversity of explanatory approaches to understanding psychosis, including social realist, behavioural, social constructionist, spiritual, cognitive and psychodynamic models, a biological understanding is the most strongly endorsed (Harlan et al., 2009). This prominent theory holds that psychosis is largely attributable to imbalances within biological pathways. This model has its origins in the Kraepelin classification system, wherein lies the inherent assumption that biological pathways will eventually map on to discrete diagnostic categories. This view has maintained a prominent and influential position in recent years, partly because of interest in gene association and family pedigree studies among those who hope to map molecular biology onto diagnosis (Corvin & Sullivan, 2016; Craddock & Owen, 2014; Lichtenstein et al. 2009; Mulle, 2012; O’Donovan et al., 2008; Straub & Weinberger, 2006).

Over time, other perspectives have developed which have sought to expand the conceptualisation of psychosis beyond Kraepelin’s medical model. Several of these were brought together in the report by Cooke (2017) ‘Understanding Psychosis and Schizophrenia’ published by the Division of Clinical Psychology. This report sought to emphasise the social factors associated with the experience of psychosis. These included income and social inequality, urbanicity, poverty, social deprivation, discrimination, overwork, poor housing and relationship problems. A key aspect of the report was the idea that there are different ways of thinking about psychosis and that each viewpoint has its advantages and disadvantages. The position of the report was that there is neither a single straightforward explanation for the etiology of psychosis, nor is there a consensus about the most helpful or effective response. The report promoted the idea of psychosis as a contentious area, a position also supported by the Critical Psychiatry Network, whose members have consistently critiqued the traditional bio-medical model (Bracken et al., 2012; Double, 2002). Supporting their argument is the failure within molecular biology research, to identify distinct defects in the structure or function of the brain as directly relating to psychosis (Moncrieff & Middleton, 2015).

Recently, there has been support from several influential figures in the field for a broader perspective on psychosis. van Os et al. (2019) argued that what are currently regarded as mental illnesses are better framed as vulnerabilities, as they are seldom ‘cured’. They critiqued the evidence-based symptom reduction model which dominates service organisations, because of its disconnection from the needs of patients. Separately, the prominent psychiatrist Sir Robin Murray publicly stated that he regretted ignoring social factors throughout his research career, and called for more research on environmental factors and epigenetics (Murray, 2017). He cautioned that those still clinging to a Kraepelinian model were refusing to accept the evidence base to the detriment of their patients. In addition, neuropsychiatric geneticists have concluded that Kraepelin’s original dichotomous diagnostic classification system now appears biologically implausible (Craddock & Owen, 2010). Despite this, the legacy of Kraepelin remains in evidence in clinical practice and research in the field of psychiatry more widely.

Considering these issues, this study aimed to use critical narrative analysis (CNA) (Langdridge, 2007) to consider what facilitates and inhibits medical professionals, with clinical experience of psychosis, to engage with the topic of psychosis as a contested area. A synthesis of rhetorical function, themes and identity work was developed across the combined narratives.

## Method

The design of the study was qualitative. To be able to participate in the study, participants had to have completed an undergraduate medical degree and the equivalent of foundation level medical training, and to have had a minimum of 6 month’s clinical experience working with psychosis. Ethics approval was granted for this study from a National Health Service (NHS) Research Ethics Committee in the United Kingdom. Approval was granted by three NHS Trusts in England, which fell within a Royal College of Psychiatry Deanery, to contact prospective participants. Full written consent was obtained from participants.

To answer the research question within the requisite time-scale for the study, sampling was purposive and aimed for representation of differentially identified views about the conceptualisation of psychosis. Deviant case analysis (Lincoln & Guba, 1985) was used to approach psychiatrists from different services, stages of career and theoretical orientations, to revise and broaden the narratives that were developing. It was decided that as a minimum, at least one participant needed to have links with the Critical Psychiatry Network, at least one participant needed to identify as a biological psychiatrist and at least one had to be between these two ends of the continuum. Prospective participants were contacted either directly via written or email correspondence, or through emails forwarded to psychiatry colleagues by clinical psychologists within the authors’ professional network.

The sample comprised four post-graduates at trainee level and eight at qualified level. A third of the sample was female. Two-thirds identified as White and the remaining third identified as Asian. Any name or narrative which might have identified someone was either altered or omitted. Data were collected through face-to-face semi-structured interviews across a nine-month period. On average, interviews were 90 minutes long and were audio-recorded and transcribed verbatim by one of the researchers.

The data analysis followed the hermeneutic circle of CNA developed by Darren Langdridge (2007). CNA was chosen because it allowed for the representation of rich and detailed data from multiple perspectives, as well as its attendance to narrative tone and rhetorical function. Although there are six specified stages, Langdridge advocates for the method to be adapted to suit individual research studies by, for example, omitting or merging stages. For this article, stages 2 and 3 were combined. Several readings of the text highlighted that identity work was woven into narratives, so separating them was less coherent. Stage 6, which is forming a synthesis, has been omitted with the understanding that the basis for this had already been completed in the preceding stages.

## Results

### Stage 1: reflexive engagement

Prior to the main narrative analysis, as part of Stage 1, the authors reflected on their own views and beliefs and how these were formed by their respective experiences in academic and clinical institutions. The authors’ training in clinical psychology had provided experience of working with a range of positions including the more orthodox positions, critical and community-based approaches. Both authors had received additional training in systemic theory and valued the emphasis within this model on multiple perspectives and social constructionism.

They recognised that they therefore approached the research with a broader perspective on psychosis than was expected from at least some of the participants. They sought to manage the influence of this stance on the gathering and interpretation of the data through supervision, reflective writing, bracketing interviewing and shared analysis. This framed each subsequent stage of the analysis.

### Stages 2 and 3: characterising narratives across a ternary of perspectives – biological, critical and less certain

Any distinct narratives, defined as having a clear beginning, middle and end, and understood to be relevant to the topic of interest within the text, were identified. The *rhetorical* f*unctions* of the narratives were considered; whether participants attempted to persuade or justify a position or imply a criticism of another. The *tone* was also attended to, which related to how stories were told and the way people constructed themselves through their narratives.

The purposive sampling demarcated two distinct perspectives, biological psychiatrists and critical psychiatrists, as well as a third more ambivalent perspective.

Those less certain in their position comprised four participants. They positioned themselves in their rhetoric as nonpartisan and unaligned to any fixed position:

Interview 1: I don’t want to dismiss something; I’m trying to incorporate the different views.

Their narratives related to how they were shaping their views by hearing speakers from the field of genetics, the Critical Psychiatry Network and The Hearing Voices Network; speaking to colleagues with different ideas; reflecting on their own personal and professional experiences and exercising critical engagement with the evidence-base. The tone of their narratives was earnest, thoughtful, reflexive and animated:

Interview 10: So, you know, quite a lot of my time is spent filling in these silly forms (laughs) that don’t benefit the patient at all. And take time away from the patient and so, I think there’s that sort of contention as well, that, some of the things that we do, eh, you know, for anyone with psychosis is, you know, not trying to serve them, but some of it is about serving the system and might even be detrimental to them in trying to serve the system.

These participants did not align themselves with any one way of understanding psychosis and recognised contentious areas they had observed and were more professionally concerned by. They referenced case examples which did not easily fit with models they were taught on training. They touched on the limited incorporation of spirituality, the statistical shortcomings of some aspects of the evidence-base and the possible collusion between pharmaceutical companies and psychiatry. They were sensitive to the personal meaning associated with psychosis, particularly within a family context.

Those who identified as biological psychiatrists comprised five participants. They were explanatory in their rhetoric and their narratives were generally brief, impersonal and canonical. The tone of their narratives was resolute and axiomatic. Any detailed narratives were about clinical case examples rather than anything personal. There was one notable exemption–a participant who engaged in narratives about his youth and journey into psychiatry. This group’s narratives focused on their high regard for what they were taught on training and read in textbooks. They constructed their professional identity as unquestionably trusting of training and unequivocal about biological reductionism, and portrayed their role as identifying symptoms and correctly diagnosing:

Interview 3: I think talking to my other general adult psychiatric colleagues, most people would say that psychosis, schizophrenia, are the most straightforward. . . the most straightforward group to treat compared to chronic anxiety, depression, personality disorders. You know exactly where you stand with them. They might not always respond to medication. They might be hugely risky (and) maybe they might be difficult to engage. But the clarity of what you’re treating and how you’ve got to treat it and what to expect in future, most general adult psychiatrists would tell you – give me psychosis any day.

Overall, these participants were either unfamiliar with alternative conceptualisations of psychosis or had not found them helpful and had not integrated them into their professional practice. This seemed to stem from an overarching belief that the genesis of psychosis is weighted towards biology over any other factor. Two participants spoke about the impact of trauma but overall, identified as biological psychiatrists:

Interview 3: That’s my belief . . . is that it’s majorly a biological illness . . . chaotic backgrounds, child-abuse, drug and alcohol use, will make it worse, there’s no doubt about that, it will exacerbate the condition, but the condition is majorly biological.

There were three participants who were more critical in their thinking. They were critically engaged in their rhetoric and in their narratives; they constructed themselves as more aware of the complications and uncertainties. They shared often deeply personal narratives and conceptualised how this influenced their engagement with psychosis. The tone of the narratives differed within this group. One participant’s narratives assumed a more reflective, compassionate tone whereas the other two were more impassioned and assertive:

Interview 5: If you go to a meeting and you declare that you’re a psychiatrist and all of a sudden people start screaming and shouting at you because they’re unhappy with what psychiatrists do, then, you know, you’ve gotta be pretty insensitive not to begin to try and understand what that’s all about.

These participants were influenced by exposure to advocacy groups, service-user movements, links to non-psychiatric academia, teaching, admired colleagues and their own life experience. They perceived research and academic psychiatry as biased towards bio-reductionism which was, in their view, having little positive impact on patients. They were often quite forthright in their views about mainstream psychiatric practice and portrayed traditional psychiatry as simplistic, crude and even potentially harmful to people. They were also more familiar with and valued alternative ways of understanding psychosis:

Interview 11: You know, and the ones that cling greatest to the reductive model, I worry about, because I think, take that doctor role away, take the status and the pay-check away – how do you function in the world? How secure are you in yourself? How, who are you as a person? And I wonder wheher there’s anyone there at all.

### Stage 4: themes of orthodoxy, vulnerability, expectation, power, populism and creativity

Themes were identified from across each of the three groups of participants through systematic reading of the text. The text was returned to repeatedly to refine themes and to explore relationships between them. There were four themes:

*1. Conventional orthodoxy*: Some participants embraced biological psychiatry and genetic vulnerability while others were more critically engaged with these ideas. Each participant spoke about anti-psychotic prescribing as being a key part of their role. Biological psychiatrists felt bio-reductive ideas represented scientific progress in their field; they were taught a lot about neurophysiology in training and continued to apply this knowledge:

Interview 6: When you treat psychosis with medication, you see instant results and we’ve seen that in practice.

In contrast, more uncertain or critical psychiatrists spoke about how this perpetuates an assumption that if something targets receptor pathways, it indicates there are imbalances in these receptors:

Interview 11: . . . the treatments for psychosis are crude and are based on very limited understanding of the human brain . . . it hardly takes a sideways look at the human experience.

*2. Invulnerability and expectation*: The position of the doctor as invulnerable and under expectation featured repeatedly as a theme. In their organisations and teams, psychiatrists described anxiety about not responding to requests to do something about a situation, with the result that they were reluctant to reduce medication dosages or use alternative approaches. This tied in with their ‘doctor’ role as synonymous with having power and not appearing uncertain or vulnerable. This identity meant having to know the answers and having to work under enormous pressure:

Interview 9: I get presented with someone and I have to do something and I have no choice, you know? The system effectively says ‘you’ve gotta do something, it’s your job. You’re the end stop. You’re the catcher’s mitt under this particular system’.

*3. Power, disempowerment and populism*: Most participants spoke of an authoritative hierarchy within the medical profession as reinforcing biological conceptualisations during training. This seemed to be perpetuated by expertise and seniority being internalised at trainee level where someone at consultant level would be someone to be deferred to:

Interview 2: Because they’re so much more an expert than you . . . you can’t really discuss things.

In addition, many participants perceived that psychiatrists who wanted to establish or defend psychiatry as a scientific specialty had greater profiles in psychiatry often with links to academia, with their views carrying more weight and status. Another factor was expectations from the wider public about what treatment patients should receive:

Interview 5: What doctors can and cannot do is quite tightly constrained – their offices, if you like, our wider social system, which has its own views about how things should be dealt with.

*4. Dampening creativity*: Participants described their training as focused on molecular biology which covered receptor pathways, incidence, genetic risk factors and treatment with anti-psychotics. The message received from training seemed to be that the dopaminergic theory was the most advanced:

Interview 4: My training would teach me that it’s excessive dopamine.

Participants portrayed a system reluctant to deviate from conventional practice; they practised within a closely scrutinised system, imbued with a fear of litigation which reinforced medical training and orthodoxy. Consumed by the demands of the sheer number of people to see, there was little space for psychiatrists to reflect upon their practice. More multi-disciplinary or holistic approaches were deemed too unrealistic because of the lack of commissioning of these approaches, the lack of time available to engage in them and their relative cost:

Interview 7: . . . We’re much more subject to audit. And I’m not saying that this is bad. To be recklessly doing whatever you like and doing negligent stuff, obviously, that’s not good. But I, you know, I think there’s a risk to sort of imagination and creativity in there somewhere.

### Stage 5: applying a critical model to the narratives

This stage assumes the position that people always speak from somewhere, from some tradition or ideology, which is inevitably limiting. By applying a critical perspective, other possibilities are highlighted. A critique was identified of the dominant paradigm in psychiatry (Bracken et al., 2012) and considered in relation to the findings. Re-examining the participants’ accounts from this different perspective provided insight into how the underlying assumptions within the dominant paradigm inhibit narratives about engagement with psychosis as a contested area.

In this study, there was a range of perspectives on what has been described by Bracken et al. (2012) as the technological paradigm, which dominates and guides psychiatry. This paradigm rests on three assumptions. First, mental health problems arise from faulty mechanisms or processes of some sort. Second, that these mechanisms can be modelled in causal terms and third, that technological interventions are instrumental and can be designed and studied independently of relationships and values.

Signs of the technological paradigm within this study were that all participants spoke about anti-psychotic prescribing as being a key part of the role; the emphasis on neurophysiology during training; the need to appear secure and certain as a medical doctor; an authoritative hierarchy; the value placed upon this paradigm at societal level and a closely scrutinised system.

Adherence to the technological paradigm limited engagement to wider questions about the conceptual nature of psychosis. Regarding psychosis as essentially biological ensured that social, relational and existential aspects of the experience were given less attention. Negative consequences were described for threatening the fundamental tenets of the paradigm, which allowed debate to be stifled and kept alternative or critical ideas on the periphery:

Interview 5: At one end of the scale, you’ve got the adherence to orthodox things, who grasp the orthodox and use it as an entrepreneurial tool to . . . the pursuit of self-advancement. Ye, being a bio-medical scientist is a good way to advance yourself and then, at the other end of the normal distribution you’ve got the awkward squad like me, who ask the difficult questions . . . but may risk professional censure as a result.

Bracken et al. (2012) contend that the technical and empirical elements of the technological paradigm should not be disavowed, but that attention to values and relationships should be primary, to develop an approach that is sensitive to the complex interplay of biological, psychological, social and cultural factors. Other prominent figures have made similar arguments (van Os et al., 2019). Prioritising the ‘human stuff’ that participants recognised could be easily overlooked by attention to technological or empirical aspects of care but it would promote greater collaboration with the service user movement, as well as facilitate closer engagement with a wider array of conceptual ideas, allowing the complexity of psychosis to be recognised and considered:

Interview 10: The randomised control trial is the gold standard of all things and ‘this is good research’ – but for me, yes, that is good research but it misses a lot of stuff that’s really important, the human stuff. It doesn’t capture any of that really. . . . But it’s really, really valuable.

## Discussion

This research intentionally focused on three groups within a sample comprised of trainee and qualified psychiatrists. The first group was biological psychiatrists who recognised few contested areas in psychosis and had little exposure to alternative ways of thinking. A second group was less certain in their thinking, and was open to having their views shaped and adapted. They recognised more areas of contention. A third group was more critical of conventional orthodox practices in psychiatry. They had more exposure to advocacy groups and were more personally reflective. The themes identified in the research pointed to an experience of disempowerment to exercise change among psychiatrists who felt that identifying with bio-reductiveness was promoted from training level upwards. A culture of invulnerability was part of the profession, reinforced by a strongly held position of power by some within it.

This research highlights the diverging, sometimes polarising views held within in the same profession. What stands out is the lack of exposure that some psychiatrists had to views which contrasted to their own. Only two of the 12 participants had read the Division of Clinical Psychology report and only four had heard of the Critical Psychiatry Network. Dialogue also seemed to be impacted by subjective hierarchy and expertise within the profession.

This hierarchy was associated with power. Senior figures in the profession, perceived as more likely to be bio-psychiatrists were considered experts whose views dominated. The Critical Psychiatry Network has commented on how power within psychiatry is both visible and invisible (Bracken & Thomas, 2001; Cutcliffe & Happell, 2009). Within this culture, participants sometimes feel compelled into conforming to mainstream psychiatric practice or adopting the idea that they are impossibly constrained by it or limited in their capacity to change it. Many of the participants’ narratives touched upon how they were overwhelmed by demands which took them away from opportunities to engage with other ideas. Of course compared to their patients, psychiatrists occupy a significantly more powerful position (McCubbin & Cohen, 1996) and a bio-reductive framework of understanding psychosis shifts the focus away from social factors (Johnstone, 2000) potentially reinforcing psychiatry’s position of power (Freidson, 2001; Moreell, 2010).

In this sample, both biological and more critical psychiatrists gravitated towards those who shared the same opinion as them, a common psycho-social tendency known as homophily (McPherson et al., 2001). The Royal College of Psychiatry expects all psychiatrists to join a peer group as part of their continuing professional development, but they are free to choose which peer group they commit to (Royal College of Psychiatry, 2015). As a result, it is likely that the different groups identified in this research would have few opportunities in a peer group setting to hear alternative views to their own.

Participants who identified as biological psychiatrists generally had narratives that were more explanatory, with interviews typically shorter than those who were more critical. One possible explanation is that less time is needed to offer a more straightforward understanding of psychosis, that it is reducible to underlying biology. In contrast, it takes more time to tell stories that require reflexivity, and to engage with uncertainty and nuance. More critical participants spoke spontaneously about their own life and inner self, something which was not anticipated before the interviews were undertaken. The person brought into the interviews, personal accounts of how they engaged with psychosis as a contested area, for example, through faith, spirituality or personal experience.

This prompts consideration about integrating reflective practice more fully into psychiatry. Currently, models of supervision in psychiatry at trainee level are often described as educational, where supervisors are allotted to oversee learning plans, goals for training and to provide feedback (Royal College of Psychiatry, 2013). Mohtashemi et al. (2016) found that psychiatrists identified numerous barriers to reflexivity including a lack of time, feeling under enormous pressure to reach quick decisions and to conform to the bio-medical model, factors consistent with this research. One apparent barrier to reflexivity is hierarchy. The psychiatrist Bekas (2013) speaks about the exceptionally hierarchical structure trainees are often faced with in medicine which extends to reflective practice, where reflections from those in a higher status are considered more valuable: ‘rules and chunks of knowledge from the “old timers” are promoted as the initiating steps to acquire legitimacy in this community’ (Bekas, 2013, p. 322).

In this research, more critical leaning psychiatrists were observed to have thought about and analysed their own actions and explored their own experiences, processes associated with reflexivity (Boyd & Fales, 1983; Stedmon & Dallos, 2009). This is turn had created new personal meaning and a change in perception of psychosis. Engaging with reflexivity can invite doubt, ambiguity and the questioning of implicit knowledge and assumptions, something which contrasts with striving to be objective, rational and unreflective.

It is apparent that there is no singular way of conceptualising psychosis, and the complexity and contentiousness of this area will undoubtedly continue. In accepting and overly identifying with one conceptual position, other possibilities become excluded. Motivation to broaden knowledge and exercise reflexivity is important and might allow for other possibilities to be considered, but is inhibited by the threat it poses to the hierarchy and power of the profession and the dominant technological paradigm.

### Limitations

Langdridge (2007) describes the CNA method as particularly demanding, ambitious and time-consuming and most previous studies using this method have been case studies. It was a significant challenge to view the relatively large group wholly while not foregoing the subtleties of each participant. Although the researchers deliberately sought differing perspectives by seeking participants from across three different Trusts, this research does not represent the views of psychiatry overall and it is possible that participants would have been more likely to agree to participate if they were more interested in the topic. Another limitation is that the researchers could have underestimated how many of the narratives elicited were impression-managed and obscured contradictions and uncertainties (Silverman, 2006).
